# The promise of growth hormone in sport: doped or duped

**DOI:** 10.20945/2359-3997000000187

**Published:** 2019-11-01

**Authors:** Ken K. Y. Ho

**Affiliations:** 1 Garvan Institute of Medical Research St Vincent’s Hospital University of New South Wales Sydney Australia The Garvan Institute of Medical Research, St Vincent’s Hospital and the University of New South Wales, Sydney, Australia

**Keywords:** Growth hormone, sports, strength, power, aerobic, anaerobic

## Abstract

Skeletal muscle is a target tissue of GH. Based on its anabolic properties, it is widely accepted that GH enhances muscle performance in sports. Athletic performance depends on muscle strength and the energy required to power muscle function. The energy required to power muscle function is derived from a continuum of anaerobic and aerobic sources. Molecular and functional studies provide evidence that in muscle GH stimulates the anaerobic and suppresses the aerobic energy system, in turn affecting power-based functional measures in a time-dependent manner. In recreational athletes, GH improves anaerobic capacity but has not been proven to significantly enhance muscle strength, power, or maximum rate of oxygen consumption. GH appears likely to selectively benefit sprint events and not physical performance that depends on strength and endurance. Arch Endocrinol Metab. 2019;63(6):576-81

## INTRODUCTION

The use of substances to gain competitive advantage in sports is probably as old as sports.

It is not known when athletes first started to use growth hormone (GH). It was promoted as a performance enhancing agent in the first edition of The Underground Steroid Handbook in 1981. This was well before the first human trials of reporting the benefits of GH in adults with GH deficiency. By then, GH had become widely known in competitive sport as a “doping agent” often used in combination with testosterone or other androgenic anabolic steroids.

The appeal of GH stems from its anabolic and lipolytic properties that increase muscle mass and reduce fat mass respectively. Evidence that GH is widely abused is evident from the number of website hits for GH supply, by customs and police drug seizures and by increasing media reports of prosecution of high-profile athletes (1). Fifty percent of athletes indicated in a survey that they would take GH if it guaranteed they would not get caught and won every competition for the next 5 years, even if they later died from adverse effects related to the drug (2).

Abuse of GH can start at a young age. A survey of 10th grade boys in the US indicated that 5% had taken GH, with more than half using GH in conjunction with steroids (3). Doses used by athletes are estimated to range from 3 to 8 mg/day for 3 to 4 days per week, often in combination with other doping agents (4), resulting in average daily doses of 1-2 mg of GH, which is approximately 2-3 times the level of daily endogenous pituitary secretion. “Polypharmacy” is widely practiced with GH most often in conjunction with anabolic steroids. A web-based survey reported that 25% of anabolic androgenic steroid users also used GH (1-10 mg/day) and insulin (5).

Due to its health risks to athletes and its potential to enhance sports performance – in addition to violating the spirit of sport – GH was listed in 2008 in the List of Prohibited Substances (http://www.wada-ama.org/rtecontent/document/2008_List_En.pdf) by the World Anti-Doping Code at all times, both in- and out-of-competition. What then is the evidence that GH enhances sporting performance? This review will cover information on the effects of GH on muscle function, on substrate and energy metabolism and examine evidence whether GH benefits different forms of physical outcome measures in recreational athletes.

## MUSCLE FUNCTION

Muscle function is regulated by many factors including genes, nutrition, lifestyle and hormones such as GH, thyroid hormones, testosterone and glucocorticoids. The stimulation of muscle protein anabolism and growth by GH has led to widespread expectation that it improves muscle strength and power. Skeletal muscles are specialized contractile tissues that control posture and physical activity while serving an important role in energy metabolism. Muscle function is dependent on the composition and strength of fibre types that require energy to drive and sustain contractile work.

Muscle function is most commonly measured as strength and power (6). Strength, which is the force generated is dependent on muscle size, type, and properties of constituent contractile proteins. Muscle power, a measure of work performed per unit time, is assessed in different ways that vary in duration. The energy required to support muscle work can be drawn from anaerobic or aerobic processes such as preformed stores or that generated from the oxidative metabolism of substrates (7). Muscle power is dependent on the availability of energy at the time of assessment. The recognition of mitochondrial myopathies as a class of functional muscle disorders arising from defects in mitochondrial respiratory chain enzymes highlights bioenergetics as an important mechanism influencing skeletal muscle function that is dependent on oxidative phosphorylation (8). The bioenergetics of muscle is an important player determining aspects of muscle function (9). These considerations are highly relevant to the understanding of the effects of GH.

## MUSCLE FIBERS

Skeletal muscle is composed of fibres that are made up of different proteins with distinct properties. Actin and myosin are functional contractile proteins, whereas tropomyosin and troponin are structural proteins that keep the contractile proteins in proper alignment giving fibres elasticity and extensibility. Myosin protein consists of two heavy chain and four light chains. Muscle fibres are classified by myosin heavy chain (MHC) isoforms mainly into two types. Type I fibres, also known as slow twitch fibres, contain an abundance of mitochondria and rely on aerobic or oxidative pathways for energy production. These fibres determine the endurance capacity of muscle. In contrast, type II fibres, also known as fast twitch fibres, generate energy from anaerobic or glycolytic pathways due to their low mitochondrial content. These fibres have high contractile force, but easy fatigability from limited energy supply. They subserve high intensity activities such as sprinting and weight lifting. There are few small human studies investigating the GH regulation of muscle fibre composition. These studies do not provide sufficient evidence supporting a role of GH in the regulation of type I or II fibres in human skeletal muscle (9).

## BIOENERGY OF MUSCLE FUNCTION

The contractile function of skeletal muscle relies on a constant supply of chemical energy. During muscle contraction, chemical energy is converted to mechanical energy that leads to movement.

**Figure 1:**
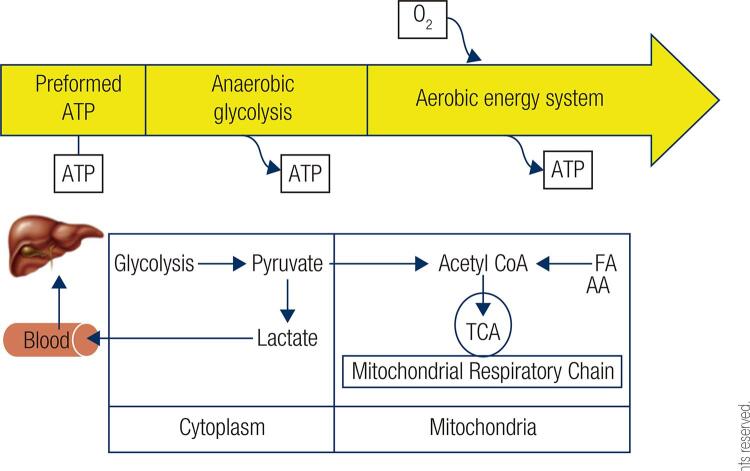
The energy continuum supporting exercise: time course of the contributions by anaerobic and aerobic energy systems in the provision of energy as ATP during exercise. AA: amino acid; FA: fatty acid; TCA: tricarboxylic acid cycle; O_2_: oxygen.

Figure 1 illustrates the metabolic processes involved in energy production and the energy continuum during physical activity. Chemical energy is available in the form of adenosine triphosphate (ATP), which is generated by anaerobic and aerobic systems (10). The anaerobic energy system relies on preformed ATP as phosphocreatine (PCr) stores and ATP production from anaerobic glycolysis i.e. breakdown of glucose in the absence of oxygen. The aerobic energy system generates ATP from oxidation of carbohydrates, lipids and proteins. In the cytoplasm, glycolysis leads to the production of pyruvate. In the absence of oxygen, pyruvate is reduced to lactate, which is released into the circulation and converted to glucose in the liver. In tissues with adequate oxygen supply, pyruvate and fatty acid (FA) are converted to acetyl CoA in the mitochondria. Acetyl CoA is oxidised via the tricarboxylic acid (TCA) cycle and the mitochondrial respiratory chain producing ATP. The amount of preformed ATP present in the muscle cells is only sufficient to sustain physical activity for the first 5-10 seconds; thereafter, anaerobic glycolysis provides energy for another 30-40 seconds, when (11).

Many factors regulate energy synthesis from substrate utilization in exercising including nutrition, metabolic hormones and the level of exercise. Whole body lipolysis during resting condition (12) and exercise (13) is stimulated by GH which increases plasma FA levels. Given that LBM accounts for the majority of substrate metabolism in the body, and muscle comprises almost 50% of total LBM, it is widely assumed that an increase in whole body lipid oxidation reflects its action on lipid utilisation in skeletal muscle. This traditional thinking was challenged by studies in rodents as well as humans, suggesting GH action is rather tissue specific. GH inhibits the expression of genes involved in lipid oxidation in skeletal muscle of rats (14). A study of metabolic gene expression in human skeletal muscle of adults with GHD indicates that GH downregulates genes governing lipid metabolism (FA transport and β-oxidation), TCA cycle activity and mitochondrial respiration (15). For example, the expression of oxoglutarate dehydrogenase and succinate dehydrogenase complex B in the TCA cycle and ATP synthase and NADH (reduced nicotinamide adenine dinucleotide) dehydrogenase in the mitochondrial respiratory chain were reduced by up to 40%. Assuming these transcriptional changes reflect effects on protein expression, these findings suggest that GH inhibits the oxidative metabolism of substrates favouring non-oxidative (anaerobic) pathways for ATP synthesis in skeletal muscle. This observation is corroborated by a study in trained cyclists, in which GH treatment was associated with increased plasma lactate levels during moderate to intense exercise compared to placebo, implying an increased rate of anaerobic disposal of pyruvate (16).

In summary, GH effects on substrate metabolism are tissue specific. Evidence suggests that GH may promote non-oxidative or anaerobic substrate metabolism in skeletal muscle for ATP synthesis, findings contrary to its effects on whole body metabolism.

## EFFECTS ON STRENGTH

Muscle strength is commonly assessed by measuring the force or torque produced during an isometric or isokinetic contraction. Isometric strength is the MVC that can be developed against an immovable object without a change in joint angle, whilst isokinetic strength is a measure of torque/force through a range of motion in which limb is moving at a constant velocity (6). This force is principally determined by fast twitch type II muscle fibres which relies on preformed ATP for energy (7).

**Figure 2:**
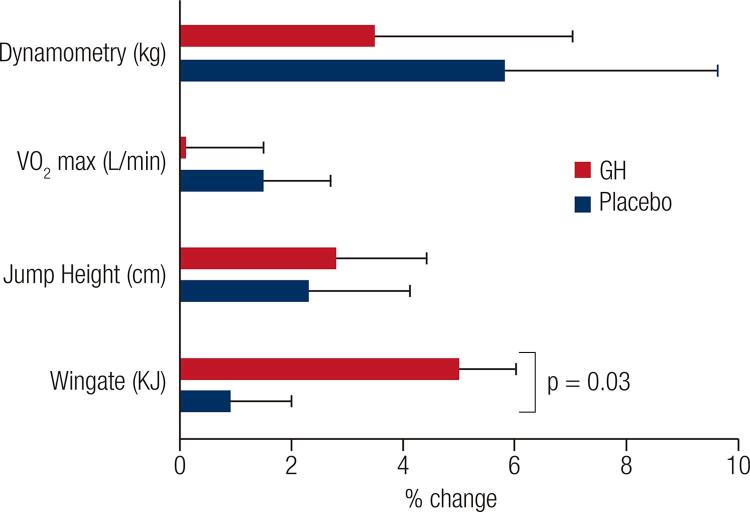
GH effects on physical performance in recreational athletes. This figure illustrates the percent change after GH or placebo treatments in 96 subjects for four measures of physical performance: VO2 max, strength (dynamometry), jump height and Wingate test. Data from Meinhardt and cols. (21).

There is strong evidence that long-term replacement of GH normalizes muscle strength in adults with GH deficiency in whom isometric and isokinetic muscle strength are reduced (9). Only a few double blind placebo controlled studies have investigated the effect of GH on muscle strength in healthy adults (17-21). A 6-week GH administration failed to demonstrate any effect on maximal muscle strength in 8 healthy males (18). Similarly, in a study of nearly 100 recreational athletes, muscle strength did not increase after 8-week of GH treatment (21) (Figure 2). GH administration in 16 healthy men combined with resistance exercise did not further enhance muscle strength more than exercise alone after 3 months (20). Studies in healthy elderly subjects have also failed to observe any increase in muscle strength following 6 months of GH therapy (17,19). These studies demonstrate that short-term GH therapy does not enhance muscle strength in healthy adults; however, the effects of long-term GH treatment are yet to be evaluated. In summary, GH increases muscle strength by increasing muscle mass in adults with GHD. At present, there is no evidence that GH enhances contractile function of skeletal muscle (22).

## EFFECT ON POWER

Muscle power is defined as work performed per unit of time and is expressed in joules per second or watts. It is described in terms of aerobic and anaerobic power, depending on which energy source is predominantly utilized to do the work. Thus, muscle power can be assessed by measuring aerobic exercise capacity and anaerobic exercise capacity.

### Aerobic exercise capacity

Aerobic exercise capacity is a measure of endurance i.e. the muscle’s ability to sustain work for prolonged period with energy provided principally from oxidation of carbohydrates or lipids in the mitochondria. In the athletic world, it determines performance in sports such as marathon, football, tennis etc., while in day-to-day life, it relates to activities such as walking. Aerobic exercise capacity is a stronger predictor of mortality in men than any other established risk factors for cardiovascular disease such as hypertension, smoking, and diabetes (23). It is determined by the measurement of maximal oxygen uptake (VO_2_ max) in L/min or ml/kg/min or maximal aerobic power output in watts or kilojoules during an incremental exercise test on a cycle ergometer or a treadmill (24).

Numerous double-blind, placebo-controlled and long-term open label trials have reported that GH replacement therapy improves and restores aerobic exercise capacity in adults with GH deficiency (22,25). The underlying mechanisms responsible for the improvement in aerobic performance during GH replacement are multifactorial. Oxygen delivery to exercising muscles depends on cardiac function, lung capacity and oxygen carrying capacity of the blood (26). Adults with GHD have impaired cardiac function, diminished lung capacity (27,28) and reduced red cell mass (29). These deficits are restored with GH replacement. However, studies show that the increase in muscle mass is associated with an increase in oxygen consumption during GH replacement (30). These observations are consistent with the delivery of a greater amount of oxygen to an increased muscle mass as a result of GH replacement in adults with GHD, leading to an increase in aerobic capacity of exercising muscles.

However, there is no convincing evidence that VO_2_ max is affected by GH treatment in healthy young adults. Based on a review of three double-blind, placebo-controlled studies assessing GH treatment in over 100 participants with doses of 2-3 mg/d, there was no treatment effect over placebo (31). The data indicate that GH supplementation in the doses used do not improve aerobic function in young healthy adults.

### Anaerobic capacity

Anaerobic exercise capacity is quantified as the total amount of work performed during a maximal exhausting exercise of a short duration that is powered by ATP supplied under anaerobic conditions (32). The Wingate test, which measures maximal power output during 30 seconds by cycle ergometry, is a widely used test for anaerobic capacity. Sporting activities that require short-term, high intensity physical activity, such as sprinting, require considerable energy support from anaerobic ATP. All physical activities including activities of daily living also depend on anaerobic energy for initiation, for the first few seconds, before aerobic metabolism kicks in as the predominant energy source (33,34). Only one study has investigated the effects of GH on anaerobic exercise capacity. This double-blind, placebo-controlled study in recreational athletes reported a significant improvement of 3.8% in anaerobic exercise capacity after GH therapy for 8 weeks, as assessed by the Wingate test (21). When translated to proportionate time reductions, the 3.8% could equate to an improvement of 0.4 second in a 10 second sprint of 100 m or of 1.2 seconds in a 30 second swim of 50 m. This improvement occurred without a significant change in body cell mass, in muscle strength and power (jump height), suggesting that muscle anabolism is unlikely to explain the improvement in sprint capacity (Figure 2). Jump height represents instantaneous work whereas the Wingate test involves all-out intensive exercise on a cycle ergometer for 30 seconds. Although both tests measure anaerobic power, the energy required for jumping is drawn from PCr stores whereas that for the longer Wingate test is derived from PCr stores and ATP derived from glycolysis. ATP generation from anaerobic glycolysis enhances the production of lactate. The finding that lactate concentrations are higher in people undergoing physical exercise after GH treatment (35) provides evidence that the anaerobic energy system is stimulated by GH. In a study by Meinhardt and cols. (21), GH treatment significantly improved sprint capacity without affecting muscle strength or aerobic capacity in the same athletes under the same conditions (Figure 2). Along with previous studies in athletes reporting that GH treatment did not improve muscle strength or endurance the collective evidence indicates that GH exerts a selective ergogenic effect on sprint capacity (22).

## PLACEBO EFFECT

A placebo effect refers to a favorable outcome arising purely from the belief that one has received a beneficial treatment (36). In their double-blind controlled study, Meinhardt and cols. evaluated the perceived and actual benefits in people allocated to placebo treatment. All participants completed a self-evaluation questionnaire which inquired whether the participant thought they were on placebo or GH treatment and how this affected their performance, without knowledge of the performance data (37). Mean perceived performance scores were higher in the subgroup who incorrectly thought they received GH compared to correct guessers. The group who thought they were taking GH displayed higher scores across all performance measures with that for sprint capacity being statistically significant. Mean changes in measured performance were higher in those who thought they were on GH that for jump height (being statistically significant. Nearly three times more men than women believed they were on active treatment (81% *vs* 31%). Compared to baseline, men who guessed incorrectly had significantly improved self-assessed scores for all categories and also increased measured performance for VO_2_ max and strength. For women, there were no significantly greater outcomes for those who guessed incorrectly compared to correctly. In short, athletes who believed they were on active treatment not only had a perceived improvement on performance, but also in measured physical performance. The effect was greater in men. This study showed that a placebo effect may contribute to perceived and actual performance-enhancing effects of GH, particularly in men (37). However, GH treatment only imparted a beneficial effect on sprint capacity compared to placebo.

## DOSE AND DURATION

The collective published information on the performance outcomes of GH treatment are limited by the dose and duration of treatment and evaluation (9). These studies employ GH doses from 15-180 μg/day for up to 12 weeks. The study that detected an improvement in sprint capacity employed a dose of 2 mg/day, approximating 28 μg/kg/day for a 70 kg person for 8 weeks. The dose corresponds to about 2-3 times daily production rates in young adults. It is possible that higher doses for longer periods may have induced a greater effect on sprint capacity or a measurable improvement in strength and endurance. Conversely the ability to detect a small effect requires a much larger sample size. It is not known what doses are used covertly for doping and the cocktails with other substances, including anabolic steroids nor their combined effects (22).

## CONCLUSION

There has been high expectation that GH enhances physical performance based on its anabolic and lipolytic actions. Physical performance in sports is a complex entity influenced by muscle size, contractile strength and the energy source required to support the duration of physical activity which in turn determines anaerobic and aerobic capacity. In fit people, GH in doses used in ethically-supervised studies does not affect muscle strength or aerobic capacity but improves anaerobic capacity. The evidence suggests that GH is unlikely to benefit power or endurance sports but likely to benefit sprint events.
